# Packing and deploying Soft Origami to and from cylindrical volumes with application to automotive airbags

**DOI:** 10.1098/rsos.160429

**Published:** 2016-09-28

**Authors:** Jared T. Bruton, Todd G. Nelson, Trent K. Zimmerman, Janette D. Fernelius, Spencer P. Magleby, Larry L. Howell

**Affiliations:** Department of Mechanical Engineering, Brigham Young University, Provo, UT 84602, USA

**Keywords:** Soft Origami, textile folding, cylindrical packing, airbag, inflatables

## Abstract

Packing soft-sheet materials of approximately zero bending stiffness using Soft Origami (origami patterns applied to soft-sheet materials) into cylindrical volumes and their deployment via mechanisms or internal pressure (inflation) is of interest in fields including automobile airbags, deployable heart stents, inflatable space habitats, and dirigible and parachute packing. This paper explores twofold patterns, the ‘flasher’ and the ‘inverted-cone fold’, for packing soft-sheet materials into cylindrical volumes. Two initial packing methods and mechanisms are examined for each of the flasher and inverted-cone fold patterns. An application to driver’s side automobile airbags is performed, and deployment tests are completed to compare the influence of packing method and origami pattern on deployment performance. Following deployment tests, two additional packing methods for the inverted-cone fold pattern are explored and applied to automobile airbags. It is shown that modifying the packing method (using different methods to impose the same base pattern on the soft-sheet material) can lead to different deployment performance. In total, two origami patterns and six packing methods are examined, and the benefits of using Soft Origami patterns and packing methods are discussed. Soft Origami is presented as a viable method for efficiently packing soft-sheet materials into cylindrical volumes.

## 1. Introduction and background

Packing and deployment of Soft Origami (soft-sheet materials folded into origami patterns) to and from cylindrical volumes is of interest in industries where a flexible sheet of material needs to fit into a confined cylindrical shape prior to being deployed via mechanisms or internal pressure.

For the purpose of this research, soft-sheet material is defined as material of constant thickness with approximately zero bending stiffness (e.g. textiles, fabrics and thin-sheet polymers). Soft-sheet materials can easily wrinkle, bend and crease locally, which can be advantageous when using them with origami fold patterns.

### 1.1. Origami and engineering design

Applying patterns extracted from or inspired by origami to engineering design can inspire new approaches to design problems. Possible benefits of applying origami to engineering design include predictable deployment, compact packing and a large number of available patterns. Previous research has explored rigid-foldable pattern applications and methods for applying origami patterns to materials thicker than paper [1–3], the use of flasher patterns for space applications [4], design of developable surfaces and other patterns based on curved-crease origami [5–7], design of deployable shelter structures [8], design of deployable cylindrical structures and masts [9–12], use of computational methods to design complex fold patterns and shapes [13,14] and general methods for selecting origami patterns and applying them to design problems [15].

Another topic that has been considered in origami and engineering is that of scalable and easily configurable packing and pattern design. If designers need to fit a common pattern (which may be scaled proportionately larger or smaller) into a specific container or packed shape, this characteristic is particularly valuable and origami provides a method for creating such scalable designs. Examples of scalable packing and pattern design within the realm of cylindrical designs include deployable solar arrays [4], heart stents [16] and a heart structure support system for patients experiencing heart failure [17].

### 1.2. Soft Origami and use of soft-sheet materials

Use of soft-sheet materials with origami patterns falls under the umbrella of ‘Soft Origami’ based on a qualitative understanding of the compliance of the soft-sheet materials being used with origami patterns. Soft Origami has recently been explored as a method for applying origami design methods to flexible materials or substrates, with potential applications in bioengineering (e.g. three-dimensional tissue scaffolding), flexible electronics and other applications where highly compliant hinges are required or useful and it is desirable to impose an origami pattern using negligible force [18]. Using origami patterns is a valuable approach due to the ability to pack soft-sheet materials into a desired packed position and subsequently deploy them from that position. Because soft-sheet materials are extremely compliant and can wrinkle, bend and crease locally to accommodate for inexact folding of the origami pattern fold lines, no strictly defined fold lines or ‘hinges’ are needed, which simplifies manufacturing. Soft Origami yields possible benefits in fields including airbag manufacturing and packing, air filter design and manufacture, parachute packing and deployment, deployable inflated structure design (including inflatable space habitats such as the BEAM module installed in 2016 on the International Space Station [19–22]), inflatable watercraft, inflatable personal amusement equipment (bounce houses, slides and hot air balloons), tent packing, umbrella packing and deployment, shade covers (e.g. track tents) and emergency blanket packing.

Deployment performance also requires consideration when applying Soft Origami to design problems. In some cases, deployment will happen many times throughout the lifetime of the product (e.g. a camping tent) and in others deployment will happen only once (e.g. automotive airbags). Depending on how critical repeat deployment performance is, different levels of care need to be taken when selecting packing patterns. In cases such as automotive airbags and parachutes where human life is dependent on the accurate unfolding of the structure, extensive testing and refinement is key to ensure that the final deployment performance meets required standards. Previous computational methods for three-dimensional airbag folding have been found successful but they are computationally expensive [23]. In other cases, many different patterns may be used to accomplish the same purpose so long as the final packed product fits in the required space or package (e.g. tent in a tent bag, umbrella in a storage bag, emergency blanket in a storage package). In applications where deployment occurs via a pressure differential (e.g. being filled with air or another gas) and where the products cannot have rigid items inside them for safety reasons (e.g. airbags), a crucial requirement which Soft Origami and soft-sheet materials can meet is a packing method wherein any rigid members used in the folding process are removed before the product is functional or deploys. This allows the design of an intentional deployment sequence (via the imposed origami pattern) while not requiring dangerous internal rigid mechanisms inside the product, and adds to the benefits of using Soft Origami and soft-sheet materials.

### 1.3. Objective

In this research, fold patterns and packing methods are considered that efficiently pack soft-sheet materials using Soft Origami techniques into cylindrical packed shapes with configurable folded (packed) height and diameter, deployed (unfolded) shape and deployed size. Deployment performance and the impact of packing method on deployment are also explored. Twofold patterns and associated packing methods each are investigated as potential solutions for packing a soft-sheet material into a desired cylindrical shape. As a demonstration, these patterns and packing methods are applied to an automotive airbag case study, and deployment performance considered and compared.

## 2. Cylindrical packing and deployment

One common current method used to pack a soft-sheet material into a cylinder is to directly compress the soft-sheet material into the desired packed shape and container without specific fold patterns (similar to how a sleeping bag is often forced into a cylindrical ‘stuff sack’). However, this technique can lead to unpredictability in deployment. Depending on the application, this may or may not be acceptable. Other basic fold patterns exist (such as the ‘tri-fold’ method commonly used to fold tents where the object is folded like a tri-fold brochure prior to rolling the material up into a tightly packed cylinder) which may be sufficient depending on the application. This research proposes two origami patterns that can be applied to soft-sheet materials to achieve a similar packed shape and size while allowing for different deployment performance, and it is shown that specific desired deployment characteristics can be dictated by modifying packing (folding) methods.

**Figure 1.**
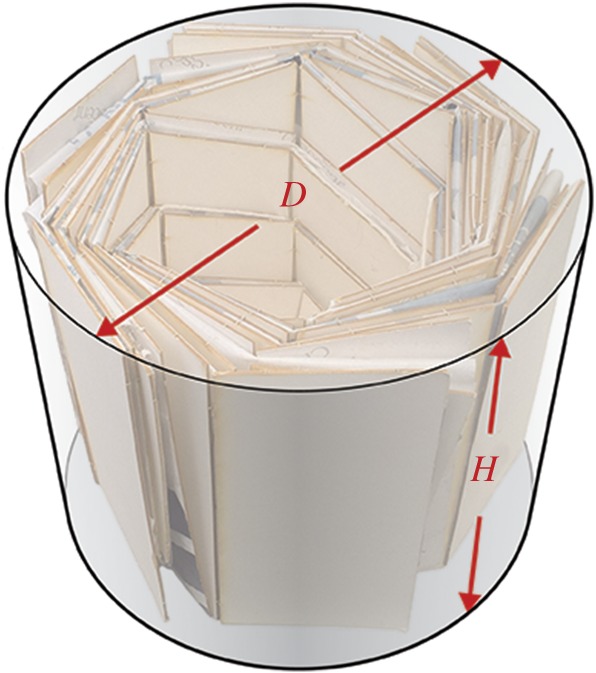
An undeployed flasher pattern with cylindrical envelope shown around it. Specified height *H* and diameter *D* are variables of interest.

The desired parameters for the final packed shape are the diameter *D* and height *H* of a cylinder circumscribed around the folded pattern, shown in figure 1. In the following subsections, our pattern selection, packing methods and deployment considerations are presented. Origami patterns are used in this research as a mechanism to generally predict packed shape and deployment characteristics.

### 2.1. Pattern selection and modelling

A search of origami patterns was conducted with a focus on finding patterns that fold into a generally cylindrical shape and can be mathematically modelled and modified. These patterns included a six-sided origami flasher model, Miura, Arc-Miura and tapered Miura [24], cylindrical Kresling pattern [10], waterbomb magic ball [25] and the fold pattern of a collapsible umbrella.

Several of these patterns do not scale well to different heights. That is, some (such as the arc-Miura) do not contract in both directions (radially and axially) when deployed. For these patterns, if the deployed structure has to be a certain packed height, the packed height is the same as that of the deployed pattern. As a result, patterns that do not scale well to different packed heights (e.g. the Miura and derivatives) were not selected for this research.

**Figure 2.**
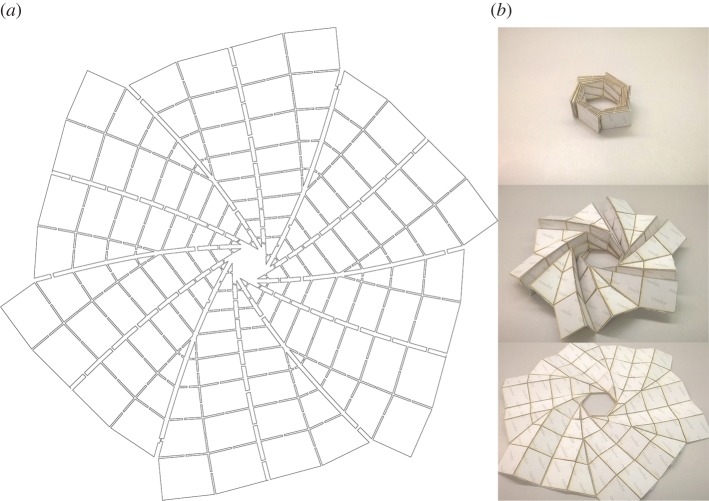
Flasher flat pattern (*a*) and matboard physical prototype with plastic backing hinge layer, in various stages of deployment (*b*).

**Figure 3.**
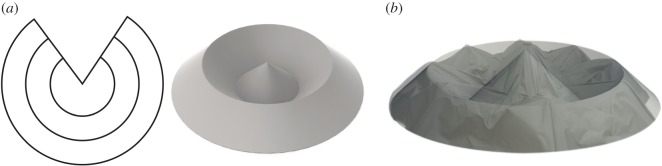
Inverted-cone fold (*a*) curved-crease origami pattern and three-dimensional model of folded pattern (*b*) umbrella photo overlaid on inverted-cone fold shape.

Two patterns were selected based on their reconfigurability and ability to pack closely into a cylinder: the flasher and the ‘umbrella fold’ patterns. The flasher pattern, shown in figure 2, is desirable due to its scalable height and deployed surface area, shape and size. Because there exist rigid-foldable versions of the flasher [26], we can create a method using rigid panel folding devices to fold a soft-sheet material into a tightly packed cylinder with the ability to select specific dimensions such as packed height and unfolded diameter. The ‘umbrella fold’, also desirable for its scalable height and configurable deployed surface area and shape, is a soft-sheet material approximation of a developable surface curved-crease pattern, the cone inversion [5,27], shown in figure 3. The developable cone inversion that exists in the umbrella fold is shown in figure 3*b* along with a semi-transparent overlay of a generic umbrella. This pattern, or variations of it, has been previously used in art pieces [28]. In particular, this is a simple version of a developable surface pattern wherein a cone is inverted several times between the peak and the base. To distinguish this particular pattern from other patterns that are used to fold umbrellas, this pattern (when folded in soft-sheet materials) will be referred to as the ‘inverted-cone fold’. The curved crease version shown in figure 3*a* has some final angle between inverted layers and cannot compress into a cylinder as a result. Using soft-sheet materials enables us to compress the pattern past the curved crease version’s final angle into a compact cylinder.

The selected patterns both allow for reconfigurable surface area, size and shape, and packed height. By changing selected parameters of each pattern, a near-infinite number of variations can be created that pack and deploy in the desired fashion. When these patterns are imposed on soft-sheet materials, the materials can be compressed into an approximately cylindrical packed shape. This feature is the most important factor used in selection of these two patterns for pursuing further. In the following section, potential manufacturing (packing) methods for applying both patterns to soft-sheet materials are explored.

### 2.2. Packing methods

Origami patterns were used as inspiration to create methods for packing soft-sheet materials into the selected patterns. Individual packing methods for each pattern are detailed as follows.

#### 2.2.1. Flasher

The flasher fold pattern, with pattern and physical prototype shown in figure 2, consists of an origami pattern that can be reconfigured to pack in a shape close to a cylinder. The example used here is a hexagonal flasher with reconfigurable height and deployed shape. Although a hexagonal pattern is shown here, other polygonal shapes are also possible. This pattern has previously been applied to space solar arrays using thickness-accommodation techniques [4] and other applications where a large deployed-area-to-packed-shape is desirable. Another benefit of the flasher is that the soft-sheet material contracts equally in the radial direction at all points around the circle of material, so there are no uneven flaps left over such as that occurs with a common collapsible umbrella.

One possible packing method for the flasher fold pattern is to use a flasher such as that made of matboard shown in figure 2 as a folding structure with the soft material on top of it; the material is folded into the flasher shape on top of the structure and then removed. A variation on this packing method is to use two folding tables with the material sandwiched in between. A second method would be to create a series of rigid links that are constrained to fold in the same method as the flasher.

#### 2.2.2. Inverted-cone fold

The inverted-cone fold pattern is shown in figure 3*a*. This is a series of cones, varying in diameter, that have been successively inverted, resulting in an array of concentric rings if viewed from above, and is also referred to as a developable surface cone inversion [29]. While the pattern shown here has only one valley and one mountain fold between the central peak and the outside edge, more rings could be added to increase the diameter of the shape. This curved-crease fold has been used for many years, particularly as art. Variations (many of which are more complex) on this pattern can be seen in Ron Resch’s work entitled ‘Yellow Cones Kissing’ as well as in multiple pieces by David Huffman such as ‘Cone Reflected 7 Times’ and pieces by Hiroshi Ogawa [6].

If the fully developable inverted-cone pattern is made of a soft-sheet material that can wrinkle, bend and crease locally, this pattern is viable for making a packed cylindrical shape where the original shape, surface size, diameter, final packed height and packed diameter are all selectable. Using material that can wrinkle, bend and crease locally allows us to use the ‘skeleton’ of the developable cone inversion shape to fold the fabric. A rigid link mechanism ‘skeleton’ can be created based on the curved crease pattern which can be used to fold soft-sheet materials into a close approximation of a cylinder. The rigid mechanism used for folding can be removed prior to deployment.

**Figure 4.**
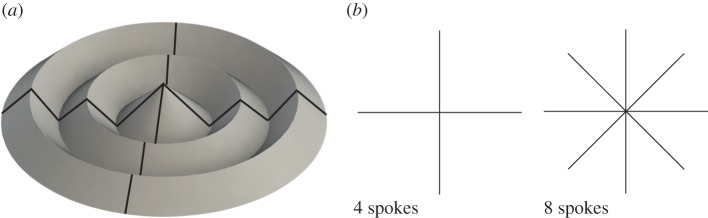
Inverted-cone fold (*a*) rigid links (similar to an umbrella) that allow an umbrella made of extensible fabric to transition from fully open to fully closed position and (*b*) two different spoke configuration options.

Several options are viable for manufacturing or folding a soft-sheet material into the inverted-cone pattern. One method was inspired by a common mechanism used to fold collapsible umbrellas. Collapsible umbrellas typically have a series of rigid links that control the fabric (a soft-sheet material). Because the fabric can wrinkle, bend and crease locally, the approximate cone inversion can go from fully packed to open, unlike a non-soft-sheet material version. Typically, an umbrella does not go completely flat when open (rather making a sloped shape to ensure that water runs off at the outside edge). The rigid links overlaid on an inverted-cone fold with two exterior rings are shown on the developable surface curved crease cone inversion in figure 4. The length of the links can be scaled to achieve a specified packed height and the number of layers dictates the deployed surface area and size. The designer can change the number of layers and layer height to achieve highly reconfigurable packed and deployed sizes. There is a trade-off between number of layers (how many rings there are viewed from the top) and packed diameter. Specifically, each ring adds at least 4*t* in overall thickness to the diameter, where *t* is the thickness of the material.

In the case of the umbrella, the links correspond to ruling lines inherent in a developable surface cone inversion [30]. If the number of links corresponding to ruling lines is increased to infinity, the lines would approach the form of a developable cone inversion surface. However, there is a physical limit due to the size and interference of the necessary link arms. Having some evenly spaced number of ‘spokes’ that are used as folding mechanisms is a potential manufacturing method, and more spokes may lead to a more accurate fold pattern, as shown in figure 4*b*.

**Figure 5.**
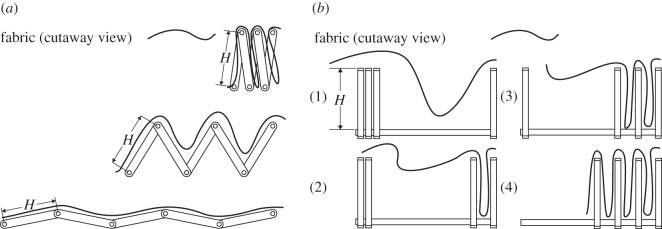
Inverted-cone fold folding methods: (*a*) linkage mechanism packing method, with fabric laid on a linkage mechanism formed of rigid links where height *H* of the packed pattern can be selected by choosing desired link lengths, and the mechanism can be removed from the material when folding is completed; (*b*) slider mechanism packing method, with fabric laid on top of the slider assembly in step 1, and folded into the layers, progressing through steps 2–4. In both (*a*) and (*b*), the final packed height *H* of the mechanism is equal to the length of the links and sliders, respectively.

**Figure 6.**
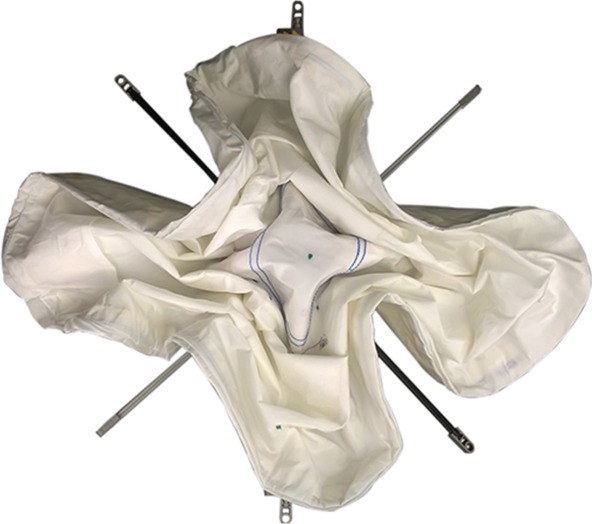
The inverted-cone fold shown in the middle of the folding process. Note the flaps of cloth that are unconstrained, which are then wrapped around the central portion to achieve the cylindrical packing.

Using the idea of spokes, two different manufacturing methods, shown in figure 5, were investigated. Each could be done with any desired number of spokes greater than three, as allowed by physical manufacturing limits. Four spokes were used in physical prototypes that were created. The first manufacturing method is to use a linkage similar to the link overlay previously shown in figure 4*a*. Figure 5*a* details this linkage, wherein fabric is laid on top of the mechanism spokes, secured using suction or weak adhesion and the linkage then folded up. In this case, the height *H* of the folded pattern will be approximately equal to the length of each link. One common mechanism that could be adapted for this purpose is a scissor linkage, which would reduce the required degrees of freedom compared with the linkage mechanism shown. Owing to the discretized nature of the spokes used in this method, there will be leftover flaps that need to be dealt with (shown in figure 6), which are minimized by increasing the number of spokes.

The second potential manufacturing method for performing the same fold is to use slider mechanisms for spokes, as shown in figure 5*b*. In this method, the fabric is pushed down between the sliders in progressive steps, with each slider constraining it against the previous (more interior) one. The height of the packed pattern will be approximately equal to the height of the sliders. This pattern will have similar leftover flaps of fabric.

One way to account for the leftover flaps resulting from both methods is to wrap them in a clockwise or counterclockwise fashion around the compacted shape, similar to a collapsible umbrella. After the folding has taken place, the folding frame can be removed from the compacted fabric before packing the fabric into a cylindrical housing by wrapping the remaining flaps around the central portion and securing the fold with a restraint.

### 2.3. Deployment rotation

When Soft Origami is used, rotation may occur during deployment. With the flasher and inverted-cone fold patterns, the main predictor of deployment rotation is whether flaps of material were wrapped in a circular pattern at any point in the packing process. In the case of the flasher pattern, as the pattern folds up, each portion of the soft-sheet material experiences rotation due to the nature of the basic flasher pattern, which can be seen in three stages of deployment figure 2. A packed Soft Origami flasher will rotate when deployed as it travels through the reverse of its folding motion. Depending on how the inverted-cone fold was packed, there may or may not be rotation. If there are leftover flaps that must be wrapped around the central portion, as shown in figure 6, there will be a corresponding rotation when deployed.

## 3. Application: automotive airbags

Driver’s side airbags have previously been packed in rectangular prism shapes and the steering wheel shape designed to fit around the airbag unit. Recent trends point towards automakers using cylindrical steering columns and cylindrical central portions of steering wheels. As a result, current fold patterns have been modified to fit cylindrical mounts instead of rectangular mounts, which has resulted in less-than-ideal use of space due to gaps around the edges. Another approach that has been successfully used is a compression fold wherein the airbag is compressed into a cylindrical shape using a cylindrical mould and thousands of pounds of applied force. A base pattern that more closely approximates a cylinder is of interest to improve packing efficiency. As such, airbags are a prime example of a soft-sheet material that needs to be folded into a cylinder and has critical deployment characteristics.

**Figure 7.**
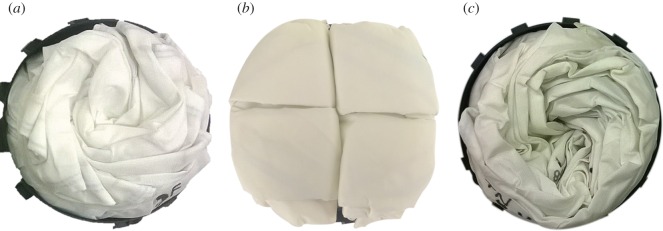
Top view of (*a*) flasher pattern imposed on an airbag in a housing, packed using a rigid folding table made of matboard and flexible hinge substrate for the flasher pattern, (*b*) baseline fold imposed on an airbag in a housing, folded using a traditional rectangular fold that has the corners compressed or forced inward in order to fit within the circular perimeter of the airbag housing and (*c*) inverted-cone fold imposed on an airbag in a housing, packed using four slider mechanisms for the inverted-cone fold pattern. All patterns were compressed to the same height prior to images being taken. Note the black housing visible under (*a*) and (*c*) but not (*b*), preliminarily indicating more efficient cylindrical packing for the flasher and inverted-cone patterns.

In collaboration with automotive airbag manufacturer, Autoliv, the inverted-cone fold and flasher fold patterns were imposed on three driver’s side airbag modules and deployed using standard test procedures. The original (baseline) fold pattern is shown in figure 7*b*, where a rectangular prism fold pattern is packed into a cylindrical shape. Both cylindrical packing efficiency and deployment performance were tested to compare the different folds with the baseline. In this application, a comparison of ‘bag pack space used’, or the height of space available for an inflator (the gas generating device, which typically is a metal cylinder) to be inserted into the back of the airbag module after packing, was also performed.

### 3.1. Packing methods applied

The aforementioned packing methods were applied to automotive driver’s side airbags consisting of two circular soft-sheet-material discs sewn together around the outer edge and having an inflator inserted through a central hole in the bottom layer of material. Nylon airbags, measuring approximately 0.25 mm in thickness per layer, were packed into a cylindrical mount with a diameter of 122 mm and height of 57 mm.

#### 3.1.1 Flasher

The folding table method was applied to the airbag to pack the flasher pattern into the cylindrical shape mentioned above. In this case, a folding table made from rigid matboard panels glued to a flexible substrate (to act as hinges along the fold lines) was created from calculated dimensions matching the desired stowed height and diameter. An airbag folded with the flasher pattern applied to it is shown in figure 7*a* with a comparison image of the baseline fold in figure 7*b*. The flasher fold shows improvement in cylindrical packing efficiency. Considering how well the flasher fold stays within the boundary of the mount, compared with the baseline fold, there is a marked improvement. The bag pack spaces were not equivalent, with the flasher fold providing 2.5 mm (10%) less bag pack space, shown in table 1. Owing to the bag pack space being worse, the flasher fold had mixed results in the requirement to fit into a cylindrical space efficiently in this particular application.

**Table 1.** Comparison of bag pack space (height of space available for an inflator to be inserted in the back of the airbag module after packing) for baseline, inverted-cone fold and flasher fold.

fold pattern	bag pack space (height, mm)	% less than baseline
baseline	26	0
inverted cone fold	25.8	0.8
flasher fold	23.5	10

#### 3.1.2. Inverted-cone fold

The slider method was applied to the airbag to pack it into a cylindrical shape, with the resulting airbag shown in figure 7*c*, alongside a comparison of the baseline fold in figure 7*b*. For this prototype, the extra flaps left over when using a four-spoke folding mechanism were wrapped in a clockwise direction around the rest of the airbag upon removal from the folding frame. Upon inspection, the inverted-cone fold shows improvement in the cylindrical packing efficiency. One comparison is to evaluate how well the inverted-cone fold stays within the boundary of the mount. In the case of the baseline fold, the outside edge of the mount is only visible in a few spots due to the folded airbag overhanging the outside edge of the shape. Furthermore, the packed height was approximately equal, with the inverted-cone fold providing only 0.2 mm (0.8%) less bag pack space, shown in table 1.

### 3.2. Deployment performance

**Figure 8.**
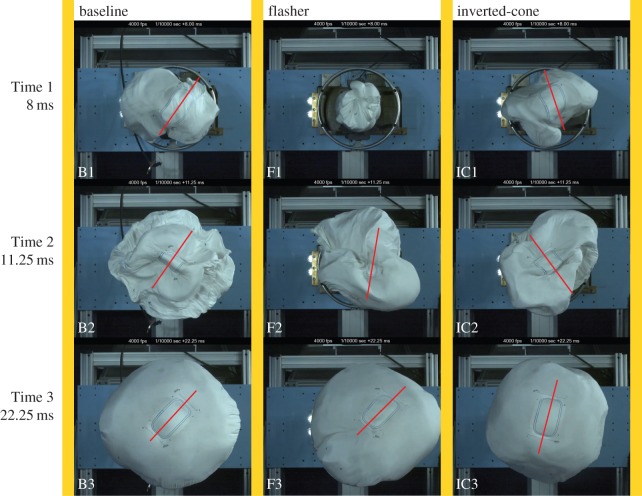
Images of threefold patterns implemented in airbags in live deployment tests. Patterns include the baseline fold, flasher fold and the inverted-cone fold. Centrelines are imposed to show orientation of central panel. Columns represent different fold patterns as labelled. Rows represent three different times: 1–8 ms; 2–11.25 ms; 3–22.25 ms. Images were taken at times when important deployment characteristics could be compared. All images were obtained in collaboration with Autoliv.

The airbag modules were tested in airbag test facilities in collaboration with Autoliv. Figure 8 shows images taken of the deployments using high-speed video. Tests were run at ambient (room) temperature and followed common deployment test protocols. Desirable deployment characteristics were noted and observed in each test. Some of the more crucial characteristics, such as the central panel of the airbag presenting itself first to the occupant as well as minimal spin when deploying, were monitored closely. To compare rotation of the airbags, centrelines were added to each photo where the central panel is visible in order to make rotational comparisons more convenient.

#### 3.2.1. Flasher

Comparing the flasher (*F*) performance to the baseline (*B*) in figure 8, at Time 1 (8 ms) there is differing deployment status in B1 (baseline pattern) and F1 (flasher pattern). F1 is still in a cylindrical packed shape and has begun to extend outwards (towards the camera) but not radially.

Continuing the comparison at Time 2 (11.25 ms), the majority of radial deployment has occurred in the baseline pattern in B2, while the flasher folded airbag in F2 has not deployed far enough radially for the central panel to be clearly visible. F2 also shows undesirable rotation, binding and whipping as it opens.

At Time 3 (22.25 ms), B3 has completed its deployment and stabilized as desired. F3 is barely fully deployed and is twisted and off-centre. Following the frames shown here, F3 did not fully stabilize until after at least 30 ms.

Analysis of the results by Autoliv personnel after multiple tests including the test shown in figure 8 concluded that the flasher pattern, while showing promising cylindrical packing efficiency, underwent approximately 180° of rotation, which is undesirable for the airbag application. It also did not deploy fast enough radially or present its central panel to the occupant as quickly as desired. For these reasons, additional work is necessary before it could be a viable fold option for application with a driver’s side airbag. As a result of its poor performance in this application, the flasher fold was removed from consideration as research moved forward.

#### 3.2.2. Inverted-cone fold

Comparing the inverted-cone (*IC*) performance to the baseline (*B*) in figure 8, at Time 1 (8 ms) we see similar deployment in B1 (baseline pattern) and IC1 (inverted-cone pattern). Both B1 and IC1 show favourable presentation of the central panel (blue rectangular outline in the centre) and similar, good deployment progression.

Continuing the comparison at Time 2 (11.25 ms), we see that the majority of radial deployment has occurred in the baseline pattern in B2, while the inverted-cone folded airbag in IC2 shows similarities but is slightly behind and is exhibiting less-than-ideal radial deployment. Specifically, B2 shows a more circular deployment while IC2 is deploying in a slightly oval shape.

At Time 3 (22.25 ms), B3 has completed its deployment and stabilized as desired. IC3 is slightly behind in its deployment, and while it has deployed fully in the radial direction (it is presenting a fully circular front panel) it can be seen that the central panel is rotated when compared with B3. This rotation shows that it is not completely stable. Following the frames shown here, IC3 did not fully stabilize until approximately 38 ms, well after the acceptable time limit for stabilization. Overall, it performed similarly to the baseline pattern but showed some unfavourable rotation and stabilization issues.

Analysis of the results by Autoliv personnel after multiple tests including the test shown in figure 8 concluded that the inverted-cone fold performed better than the flasher. It showed good radial deployment and promising deployment speed, and performed the closest to the baseline pattern. However, it did show an unwanted 90° twist which should be minimized before it can be implemented in commercial airbags. One cause of this twist is that the folding process results in multiple portions of the bag not packing into the original cylinder (flaps that extend out after the slider or slider link mechanisms are used to compress the material to the centre) and instead needed to be wrapped around the central shape to fit (thus leading to a spin when unwrapping). This is analogous to wrapping the remaining cloth around a collapsible umbrella when the arms are folded in and then using a strap to secure the wrapped cloth. Figure 6 shows an image of an airbag at this point in the folding process. Based on the promising performance and evaluation of the inverted-cone fold, research moving forward focused solely on improving the performance of this pattern.

### 3.3. Packing method modification based on deployment performance

To address deployment performance, new packing methods were created for the inverted-cone fold to minimize rotational problems. The patterns remain the same, but new methods can be used to achieve performance closer to the desired function of this particular application. While the following methods can be used to improve the airbag deployment performance, other methods may be more appropriate for different applications. One of the main benefits of using Soft Origami is that the same base pattern can be used with different packing methods to achieve different deployment performance.

**Figure 9.**
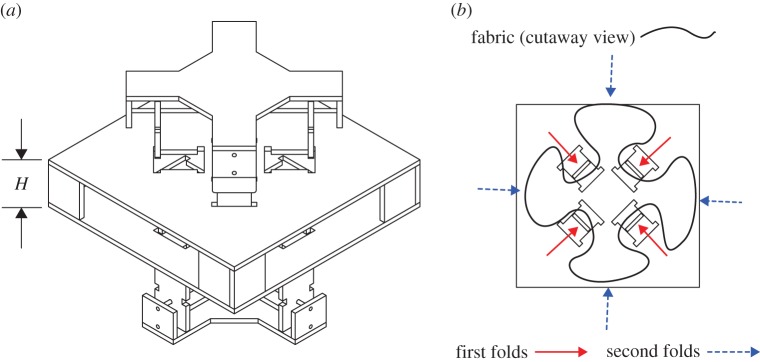
Offset cross mechanism and fold sequence, with (*a*) isometric view of offset cross mechanism. Height *H* of the packed pattern can be selected by choosing desired offset between top and bottom panels. Note two sets of cross-shaped mechanisms, each with four slider mechanisms, with the top cross offset 45° from the bottom, which are alternately inserted into the panels from the top and bottom and used to pack the inverted-cone fold pattern. In (*b*), we see a top view of a sample fold sequence, where the first set of folds are imposed in one direction and then the second set of folds are offset 45°, thus increasing the discretization of the fold and reducing the size of the leftover flaps.

Two new packing methods were created to pack the inverted-cone pattern in a way that reduces rotational deployment spin. The first new packing method is called the offset cross method and it aims to increase the discretization of the folds, that is, to increase the number of spokes (mentioned in §2 and shown in figure 4*b*). In this case, eight spokes were used to decrease the size of the flaps, thereby minimizing the level of resulting deployment spin. The apparatus for this method consists of two cross mechanisms and two panels shown in figure 9*a*. In this method, the airbag is laid between the panels and the first cross is inserted from the bottom into the bottom slots to generate the first fold. The cross is then actuated to compress the fold towards the centre. The second cross is then inserted from above into the opposite panel at a 45° offset, and actuated using the same process. The first cross is then removed, and the process is repeated until the airbag is completely folded. Figure 9*b* further depicts the offset nature of the panel slots, and the motion of the cross paddles.

**Figure 10.**
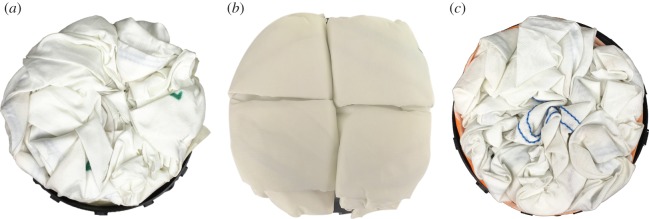
Top view of (*a*) inverted-cone fold imposed on an airbag in a housing, packed using the described offset cross method for the inverted-cone fold pattern, (*b*) baseline fold imposed on an airbag in a housing, folded using a traditional rectangular fold that has the corners compressed or forced inward in order to fit within the circular perimeter of the airbag housing and (*c*) inverted-cone fold imposed on an airbag in a housing, packed using the described nested cylinder method for the inverted-cone fold pattern. All patterns were compressed to the same height prior to images being taken. Note the black housing visible under (*a*) and (*c*) but not (*b*), preliminarily indicating more efficient cylindrical packing for the inverted-cone fold offset cross and nested cylinder packing methods.

This method was developed primarily for its ability to decrease flap size, but also because of its ability to be automated. Eight wide paddles (above and below) are re-used for each fold, which significantly decreases the number of degrees of freedom of the folding apparatus. The inverted-cone fold was applied to an airbag of the same style, size and material as that used in the baseline. This is shown in figure 10*a* alongside a comparison of the baseline fold in figure 10*b*. For this prototype, the leftover flaps on the right-hand side were wrapped clockwise, while the flaps on the left were wrapped counterclockwise. Note that this method is only a discretized version of the inverted cone fold, and with more paddles it would yield a final packed state that more closely resembles the inverted cone fold, and additionally would have a smaller flap size. The packing efficiency was similar to that of the baseline, but is expected to be improved with a well-developed folding apparatus.

**Figure 11.**
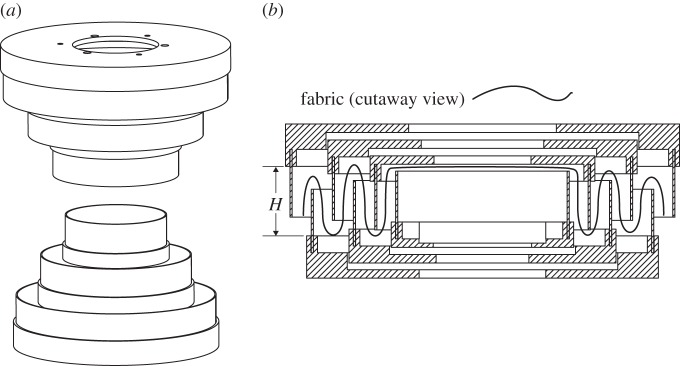
Nested cylinder mechanism and fold sequence, with (*a*) isometric view of nested cylinder mechanism. Note multiple nested cylinders, which are inserted from alternate directions from small to large, imposing one layer of fold at a time. In (*b*), we see a cutaway view of the final fold configuration, with height *H* determined by height of interior cylinder, with the mountain and valley folds imposed on the airbag. A cinching belt is then placed about the outer cylinder, and is tightened as each successive cylinder is removed from large to small. This method evenly distributes all remaining flaps.

The second new folding method is called the nested cylinder method, and it aims to eliminate the extra flaps altogether by using continuous folds. The apparatus for the nested cylinder folding method consists of multiple cylinders, which are placed alternating from above and below (figure 11*a*). A cinching belt (made of a flexible sheet of polymer, textile, alloy, etc.) is also included. To fold, the material is placed over the first cylinder. A second cylinder with a larger diameter is brought down around the first cylinder, pushing a fold into the material. Successive cylinders are introduced in a like manner, from alternating directions, until the airbag is completely enclosed. The cinching belt is then placed around the outermost cylinder, and is tightened as each cylinder from large to small is removed. Figure 11*b* further depicts how the folds are created as cylinders are added, and this method results in the highest-fidelity approximation of the original inverted-cone fold curved-crease pattern when compared with the other methods.

This method is also easily automatable, and has the potential of using mechanisms with fewer degrees of freedom than the offset cross method. A concern with this method is the friction between the cylinder edges and the fabric, but this can be mitigated with a refined surface finish and rollers placed around the edge of the cylinder. An airbag folded with the inverted-cone fold applied to it is shown in figure 10*c*, alongside a comparison of the baseline fold in figure 10*b*, showing similar improvement to previous patterns at packing into a cylindrical housing.

### 3.4. Deployment performance for new packing methods

**Figure 12.**
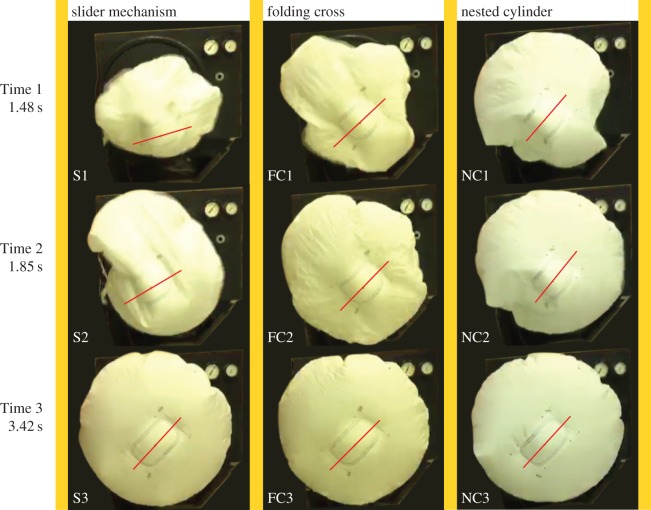
Images of three packing methods for the inverted-cone fold implemented in airbags in test stand deployment tests. All deployments were done using compressed air and deploying at about one-fifteenth of the speed of the prior deployment tests. Packing methods include the slider mechanism (using four sliding mechanisms, the same method and pattern shown previously in figure 8 under the title ‘inverted-cone’) as a baseline as well as the folding cross method and nested cylinder method. Centrelines are imposed to show orientation of central panel. Columns represent different packing methods, as labelled. Rows represent three different times: 1–1.48 s; 2–1.85 s; 3–3.42 s. Images were taken at times when important deployment characteristics could be compared and are at approximately the same proportional times throughout deployment as prior images. The folding cross method and nested cylinder method appear to show less rotation upon deployment.

Airbags were deployed at Brigham Young University to test the folding methods described above, with resulting images shown in figure 12. Three flap treatments were also tested for the original slider mechanism: increasing from four to eight spokes (resulting in smaller flaps), clockwise and counter-clockwise folded flaps, and map-folded (reversing pleats rather than being wrapped all in one direction) flaps. The deployments were filmed at a rate of 60 frames per second, on a test stand that uses compressed air to inflate the airbag (resulting in a deployment at about one-fifteenth of the speed of actual airbag inflators). Owing to the difference in deployment method, further live deployment tests using standard test equipment and deployment speed would be necessary to directly compare these results with those obtained in collaboration with Autoliv and shown in figure 8. To determine whether the inverted-cone fold performed similarly in both tests (and thus if the tests are likely to show similar performance), an airbag folded using the same method and pattern was deployed and is included in figure 12, labelled ‘slider mechanism’. It was seen in both cases that the angular rotation of the central panel between Time 1 (8 ms and 1.48 s, respectively) and Time 3 (22.25 ms and 3.42 s, respectively) was similar (albeit in the opposite direction, probably due to the leftover flaps being wrapped in the opposite direction), indicating that the compressed-air tests should show similar deployment characteristics. Each deployment was evaluated for rotational spin, and it was concluded that all of the different flap folding techniques, as well as the two airbag fold methods described previously (offset cross and nested cylinder), decreased deployment spin. To compare rotation of the airbags, centrelines were added to each photo where the central panel is visible in order to make rotational comparisons more convenient.

Examining the results presented in figure 12, we see that at Time 1 (1.48 s) the slider mechanism (S1) is still opening up and rotating clockwise, while the folding cross and nested cylinder (FC1 and NC1, respectively) are more stable and further along through deployment. At Time 2 (1.85 s), all three are nearly fully deployed radially, and we can see that the central panel (rectangle) of FC2 and NC2 are still aligned with their previous deployment photo and are more stable than S2. Finally, at Time 3 (3.42 s) we see that all are fully deployed and stable. Both the folding cross method and nested cylinder method exhibit nearly zero rotation throughout deployment (as seen by the location of the central panel as well as the clarity of the photos indicating little movement), while the slider mechanism (the fold titled ‘inverted-cone fold’ previously discussed in §3) exhibits a significant (greater than 30°) rotation.

## 4. Discussion and conclusion

In this paper, fold patterns and packing methods have been introduced and evaluated to efficiently pack soft-sheet materials into cylindrical packed shapes with configurable folded (packed) height and diameter, deployed (unfolded) shape and deployed size. Deployment performance and the impact of packing method on deployment was also explored. Twofold patterns (the flasher and the inverted-cone fold) and a total of two packing methods for the first pattern and four for the second pattern were presented as viable solutions. Application to automotive airbags was explored and results showed promise, although the flasher was shown to be less-than-ideal for driver’s side airbags and would probably be more valuable in other Soft Origami applications.

Both fold patterns have adjustable stowed height and diameter, deployed shape and deployed size while folding into approximately cylindrical shapes. We were able to influence the behaviour of the airbag using this approach, and preliminary testing showed that we were able to specify packed behaviour, unfolding behaviour via pressure difference deployment and final deployed shape. Both patterns showed favourable improvements in packing an airbag into a cylindrical shape with sufficient room underneath the packed material for an inflator.

Multiple possible methods were created and explored to fold the inverted-cone fold and flasher fold patterns using a rigid frame that is later removed. After frame removal, the folds are ready to be deployed by way of a pressure differential. The patterns, shown through an application to automotive airbag folding, accomplished the desired goals of the research. The packing methods demonstrated here have been shown to work when folded by hand (with a combination of a mechanism and human intervention), but have not yet been automated, which could be a topic of further work.

Another accomplishment of this research was the modification of packing method based on deployment performance. Although the same pattern (the inverted-cone fold) was used, different packing methods were shown to influence deployment performance, which is probably true of many Soft Origami patterns and applications. That is, unlike traditional origami, where fold lines constrain behaviour, Soft Origami allows for a more quantitative approach wherein the same pattern can be packed using many different methods (with varying fidelity to the original pattern) depending on the application constraints. However, different packing methods and levels of discretization result in packed patterns that match the original desired pattern with varying degrees of fidelity.

In conclusion, multiple patterns and packing methods were presented that are well-suited for packing a soft-sheet material into a cylindrical volume prior to deployment via internal pressure. Another unique development in this work is the use of an origami-pattern-inspired folding frame to impose the pattern on the soft-sheet materials, and then removing the folding frame and maintaining the folded shape for use in deployment via pressure difference (e.g. inflation). This is advantageous for a mechanism or structure that would present a safety hazard to humans if it had a rigid understructure when deploying. In an application to automotive airbags, we also demonstrated the principle of modifying the packing method (within the same origami fold pattern) based on deployment performance and requirements.

## Data accessibility

All data used in this research is included in figures and table.
